# Tumor size, TIRADS, and lymph node status as predictors of central neck dissection in thyroid cancer

**DOI:** 10.11604/pamj.2025.52.39.48016

**Published:** 2025-09-25

**Authors:** Mohammed Al-Essa

**Affiliations:** 1 Department of Otolaryngology-Head and Neck Surgery, College of Medicine, King Saud University, Riyadh, Saudi Arabia

**Keywords:** Thyroid cancer, central neck dissection, tumor size, TIRADS, lymph node metastasis

## Abstract

**Introduction:**

the aim was to evaluate the predictive value of tumor size, TIRADS classification, lymph node involvement, and demographic factors in guiding central neck dissection (CND) in patients with thyroid cancer.

**Methods:**

this retrospective cross-sectional study included 437 patients, selected through convenience sampling, who underwent total thyroidectomy for thyroid cancer at a tertiary hospital in Riyadh, Saudi Arabia, from 2010 to 2023. Data collected included age, sex, BMI, tumor size, TIRADS level, lymph node status, and fine-needle aspiration (FNA) results. Associations with the performance of CND were analyzed using Pearson correlation coefficients and descriptive statistics.

**Results:**

lymph node involvement, defined as histopathologically confirmed central lymph node metastasis, was the strongest independent predictor of CND (r = 0.5285, p < 0.0001), followed by tumor size (r = 0.4200, p < 0.0001) and TIRADS score (r = 0.2128, p = 0.0467). Age showed a weak but statistically significant association with CND (r = 0.1426, p = 0.0031), while sex and BMI had no significant impact. The majority of nodules were classified as TIRADS 3 to 5 (87.17%), and patients undergoing CND tended to be older with larger tumors and higher TIRADS categories.

**Conclusion:**

lymph node involvement, tumor size, and TIRADS classification are meaningful predictors of surgical management in thyroid cancer. Integrating these factors into preoperative evaluation supports a personalized approach to CND, potentially reducing unnecessary procedures and improving patient outcomes. These findings support the development of standardized, risk-adapted surgical criteria for central neck dissection in thyroid cancer.

## Introduction

Thyroid cancer is the most common malignancy of the endocrine system, and its global incidence has shown a marked increase over recent decades. In the United States, it remains the fastest rising cancer by incidence, with over 52,000 new cases estimated annually as of 2020 [1]. Despite its typically favorable prognosis, outcomes are highly dependent on several clinicopathological factors, including tumor size, histologic subtype, and lymph node involvement. The presence of lymphatic spread, in particular, significantly raises the risk of recurrence and may influence long-term survival [2].

Central neck dissection (CND), which involves the removal of lymph nodes within the central compartment of the neck, is often performed in patients with papillary thyroid carcinoma (PTC) the most prevalent form of differentiated thyroid cancer. Lymph node metastasis is common in PTC and may be present even in early-stage disease [3]. However, while CND has been shown to potentially reduce recurrence rates, it carries a risk of considerable morbidity. Complications such as recurrent laryngeal nerve injury and hypoparathyroidism remain major concerns and highlight the necessity of selecting appropriate candidates for the procedure [4]. Although the American Thyroid Association (ATA) has proposed clinical guidelines for CND, these recommendations are not universally applied, often resulting in inconsistent surgical practices [5].

Several parameters have been proposed to help guide the surgical approach, including tumor size, cytologic findings on fine-needle aspiration (FNA), and radiologic risk stratification through the Thyroid Imaging Reporting and Data System (TIRADS). Larger tumors are more likely to harbor nodal metastases and may require more extensive surgical management [6]. FNA is widely regarded as the standard for preoperative cytological evaluation, yet discordance between FNA and final histopathological diagnosis can complicate decision-making [7]. Meanwhile, the adoption of TIRADS classification has improved the identification of nodules with high malignant potential through ultrasound-based criteria, allowing for more precise preoperative risk assessment [8].

In addition to tumor-based features, patient demographics such as age, sex, and body mass index (BMI) are increasingly recognized as potential modifiers of disease progression and surgical risk. Older patients and males are often reported to exhibit more aggressive forms of thyroid carcinoma, whereas elevated BMI has been linked with increased tumor burden and a higher likelihood of postoperative complications [9,10].

Despite these findings, there remains a lack of integrative models that combine these predictors to guide individualized CND decision-making. There remains a lack of standardized criteria that integrate tumor characteristics, radiological data, cytologic findings, and demographic variables into a unified model to inform surgical planning. This gap has led to variability in practice and potentially unnecessary surgeries that may expose patients to avoidable risks.

The present study aims to evaluate the clinical factors that influence the decision to perform central neck dissection in patients with thyroid cancer. Specifically, we analyze the correlation of tumor size, lymph node involvement, TIRADS category, FNA results, and demographic features with final histopathological outcomes. By doing so, we hope to refine the selection criteria for CND, reduce surgical morbidity, and contribute to more personalized, evidence-based thyroid cancer management.

## Methods

This study aimed to evaluate the indications for central neck dissection (CND) in patients diagnosed with thyroid cancer by analyzing the correlation between tumor characteristics, demographic factors, lymph nodes involvement and fine-needle aspiration (FNA) findings with final histopathology outcomes. The methodology involved a comprehensive approach, which included data collection, analysis, and the establishment of evidence-based guidelines for clinical decision-making regarding CND.

**Study design:** this study employed a retrospective cross-sectional design to evaluate the clinical and radiological factors associated with the decision to perform central neck dissection (CND) in patients diagnosed with thyroid cancer. The design involved reviewing and analyzing existing clinical and radiological data at a single point in time, without follow-up, which is appropriate for identifying associations between tumor characteristics and surgical decisions. Medical records from a tertiary referral hospital in Riyadh, Saudi Arabia, covering the period from January 2010 to December 2023, served as the primary data source. A total of 437 patients were included. The sample size was not based on formal statistical calculation; instead, all eligible patients with complete data available in the electronic medical record during the study period were included (convenience sampling). All reporting followed the STROBE guidelines for observational studies.

**Study population:** the study included 437 patients of all ages who were diagnosed with thyroid cancer and underwent total thyroidectomy during the defined period. Inclusion criteria were: (1) confirmed thyroid malignancy by histopathology; (2) availability of complete clinical, imaging, cytology, and histopathological data; and (3) preoperative evaluation by fine-needle aspiration (FNA) and ultrasound imaging with TIRADS classification. Exclusion criteria included: (1) incomplete or missing medical records; (2) history of previous thyroid surgery; and (3) coexisting malignancies that could confound surgical decision-making.

**Data collection:** patient data were extracted from electronic medical records and included the following variables: age, sex, body mass index (BMI), tumor size, lymph node involvement, FNA results, TIRADS score, and final histopathology findings. These variables were specifically selected because they represent established or potentially relevant predictors of central neck dissection according to prior literature and clinical practice guidelines. All tumor sizes were documented based on histopathological measurements following thyroidectomy. Lymph node status was determined through surgical reports and postoperative pathology. TIRADS scores were collected from preoperative ultrasound reports interpreted by board-certified radiologists. All data were reviewed, verified, and cross-checked by two independent researchers to ensure accuracy and minimize extraction bias.

**Data analysis:** descriptive statistics were performed to summarize the demographic and clinical characteristics of the study cohort (i.e., age, sex, BMI). Frequency counts, percentages, means, and standard deviations were reported for tumor size, TIRADS category, FNA findings, and lymph node status, when appropriate. To fulfill the objectives of the study, Pearson correlation coefficients were calculated to evaluate the strength of association between tumor size, TIRADS scoring, lymph node status, FNA findings, and demographic variables (i.e., age, sex, BMI) with the performance of central neck dissection (CND). The strength of correlation was interpreted in accordance with standard ranges (r < 0.2 = very weak, 0.2-0.39 = weak, 0.4-0.59 = moderate, 0.6-0.79 = strong, r &#8805 0.8 = very strong). A p-value < 0.05 was interpreted as statistically significant. All analysis was performed using SPSS version 26 (IBM Corp., Armonk, NY, USA).

**Ethical considerations:** this study was conducted in accordance with the ethical principles outlined in the Declaration of Helsinki. Ethical approval was obtained from the Institutional Review Board of the King Abdullah International Medical Research Center (IRB No: NRC21R/364/09; Approval Date: September 13, 2021). As this was a retrospective study involving de-identified patient records, the requirement for informed consent was formally waived by the IRB.

## Results

A total of 437 patients with histologically confirmed thyroid cancer who underwent total thyroidectomy were included in the analysis.

**Demographic profile:** the majority of patients were female (79.86%), and the median age reflected a mid-life predominance. Most patients were aged between 31 and 60 years, with 24.03% in the 51-60 age group, followed by 21.74% aged 31-40, and 21.51% aged 41-50. Regarding body mass index (BMI), most patients were either overweight (26.32%) or classified as Obesity Class 1 (27.69%). Fewer individuals fell into the normal weight category (18.76%) or Obesity Classes 2 and 3. Only 1.37% were underweight.

**Fine-needle aspiration results:** all patients had preoperative FNA cytology. The majority were classified as Bethesda V or VI (suspicious or malignant), which was consistent with the diagnosis of differentiated thyroid carcinoma. The lower Bethesda categories (III and IV) made up a minority, meaning the cytology was indeterminate, but suspicious enough to undergo surgical management (Table 1).

**Table 1:** lymph node involvement

LN	Frequency	Percentage
0	238	54.46
1	126	28.83
9	73	16.71
Total	437	100%

LN = 9 denotes extensive or multifocal central compartment nodal metastases as defined by institutional surgical coding.

**Lymph node involvement:** among the 437 patients, 238 (54.46%) had no histopathological evidence of central lymph node metastasis (LN = 0). Lymph node positivity was observed in 199 patients (45.54%). These included 126 patients (28.83%) with limited nodal involvement (LN = 1) and 73 patients (16.71%) categorized as LN = 9, which in the institutional coding system referred to extensive or multifocal nodal metastasis involving multiple central compartment zones (Table 1).

**TIRADS classification:** preoperative ultrasound assessment revealed that 87.17% of patients had thyroid nodules classified as TIRADS 3, 4, or 5, indicative of intermediate to high risk of malignancy. Specifically, TIRADS 4 was the most frequent category (38.44%), followed by TIRADS 5 (25.40%) and TIRADS 3 (22.88%). Lower-risk categories (TIRADS 0-2 and 6) accounted for the remaining 12.83% of cases (Table 2).

**Table 2:** distribution of TIRADS categories among thyroid nodules

TIRADS	Frequency	Percentage
0	5	1.14
1	19	4.34
2	32	7.32
3	100	22.88
4	168	38.44
5	111	25.40
6	3	0.69
Total	437	100%

Higher TIRADS scores indicate increased suspicion of malignancy

**Final histopathology:** histopathological examination confirmed that the majority of cases were papillary thyroid carcinoma (PTC, 396 cases, 90.6%), followed by follicular thyroid carcinoma (FTC, 27 cases, 6.2%). Non-differentiated histologies, including medullary (n = 6), poorly differentiated (n = 3), and anaplastic thyroid carcinoma (n = 1), along with a few unclassified cases (n = 4), were excluded from the analysis to ensure focus on differentiated thyroid carcinoma (Table 3).

**Table 3:** distribution of final histopathology among the study cohort

Histopathology	Frequency (n)	Percentage (%)
Papillary thyroid carcinoma (PTC)	396	90.6
Follicular thyroid carcinoma (FTC)	27	6.2
Excluded cases (MTC, PDTC, ATC, unclassified)	14	3.2

final histopathological diagnosis of thyroid cancer cases in the study cohort

**Figure 1:**
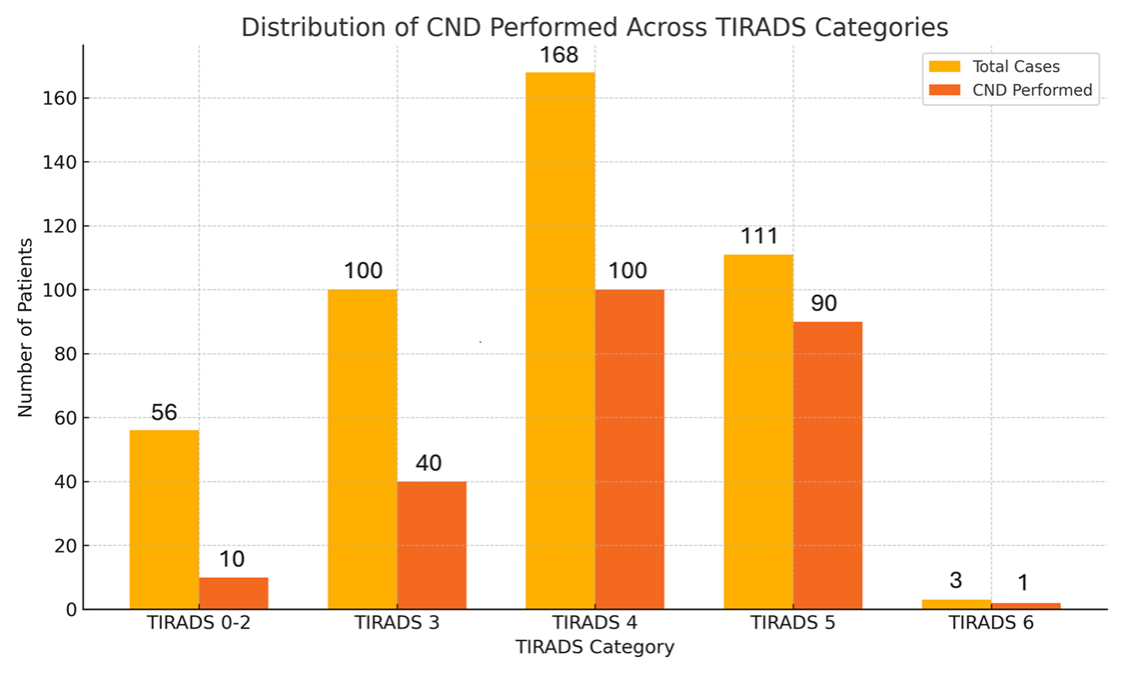
proportion of central neck dissection across TIRADS categories; TIRADS 4 and 5 nodules showed higher rates of CND, indicating a correlation between ultrasound risk category and surgical decision-making

**Association Between TIRADS category and CND:** Figure 1 illustrates the number of thyroid cancer patients in each TIRADS category (0-6) and the corresponding frequency of central neck dissection (CND) performed. A higher proportion of CND procedures was observed among patients with TIRADS 4 and 5 nodules, indicating that intermediate to high-risk ultrasound features were associated with more aggressive surgical management. This supports the clinical utility of TIRADS in preoperative risk stratification and surgical planning.

**Correlates of central neck dissection:** Table 4 summarizes the strength and significance of associations between patient factors and the performance of central neck dissection (CND). Lymph node involvement demonstrated the strongest correlation with CND (r = 0.5285, p < 0.0001), supporting its role as the primary surgical determinant. Tumor size was also significantly associated with the likelihood of CND (r = 0.4200, p < 0.0001), reflecting the clinical practice of offering more extensive surgery for larger lesions. TIRADS classification showed a moderate but statistically significant association with CND (r = 0.2128, p = 0.0467), reinforcing its utility in surgical planning. Age had a weak but meaningful correlation with CND (r = 0.1426, p = 0.0031), suggesting that older patients may be slightly more likely to undergo CND. Sex and BMI, however, showed no significant association with the decision to perform CND (r = 0.1300, p = 0.2966 and r = 0.1290, p = 0.2096, respectively), indicating that these demographic factors had limited influence on surgical decision-making in this cohort.

**Table 4:** correlation to central neck dissection surgery

Profile	Correlation	P-value	Interpretation
Age	0.1426	0.0031	weak significant association
Sex	0.1300	0.2966	no significant association
BMI	0.1290	0.2096	no significant association
Lymph Nodes	0.5285	<0.0001	moderate significant association
Tumor size	0.4200	<0.0001	moderate significant association
TIRADS	0.2128	0.0467	moderate significant association

Degree of association can be interpreted as: 0 <= r < 0.2: very weak association; 0.2 <= r < 0.4: weak association; 0.4 <= r < 0.6: moderate association; 0.6 <= r < 0.8: strong association; r >= 0.8: very strong association.

**Comparative analysis of central neck dissection vs. non-central neck dissection patients:** among the cohort, central neck dissection was performed either electively (prophylactic) or therapeutically when preoperative imaging (ultrasound or CT) demonstrated suspicious nodal involvement. In this series, 25 patients underwent elective CND, while 74 patients underwent therapeutic CND. To further contextualize the correlation findings, a descriptive comparison was made between patients who underwent central neck dissection (CND) and those who did not. Patients who received CND tended to have larger tumors, higher TIRADS categories (predominantly 4 and 5), and were slightly older on average. These observed trends support the association between tumor burden, radiologic suspicion, and surgical decision-making in thyroid cancer.

**Summary of key trends:** the data revealed that nearly half of the patients had nodal metastasis, a high proportion had intermediate-to-high TIRADS scores, and larger tumor size was closely associated with both FNA results and final histopathology. Collectively, these findings support the relevance of combining radiologic, cytologic, and clinical parameters when considering CND in thyroid cancer surgery.

## Discussion

**Principal findings:** this study provides important insights into the clinical and radiologic predictors associated with central neck dissection (CND) in patients with thyroid cancer. The most significant predictor of CND was central lymph node involvement, which aligns with prior literature emphasizing its role in recurrence risk and long-term oncologic control. Tumor size and TIRADS classification were also moderately associated with the decision to perform CND, indicating their relevance in surgical planning [10].

**Interpretation in context:** in our series, therapeutic CND was more common (74 patients) than elective CND (25 patients), reflecting the selective use of prophylactic dissection. This distribution is in line with ATA recommendations, which reserve prophylactic CND for patients with high-risk features but no radiological evidence of nodal disease. Tumor size showed a moderate correlation with CND, supporting previous findings that larger tumors are more likely to exhibit extrathyroidal extension or occult nodal disease and thus warrant more aggressive surgical intervention [11].

TIRADS classification also demonstrated predictive value. Most nodules (87.17%) were classified as TIRADS 3-5, with CND more frequently performed in higher categories, particularly TIRADS 4 and 5. These results support the utility of ultrasound-based risk stratification in preoperative surgical decision-making. Our findings mirror earlier work validating TIRADS as a noninvasive tool to predict malignancy and guide intervention.

Age showed a weak but significant association with CND, possibly reflecting greater histological aggressiveness in older patients or more conservative treatment thresholds. On the other hand, sex and BMI were not significantly associated with the performance of CND. Although male sex and obesity have been previously associated with more aggressive tumor features, our data suggest these factors alone do not substantially alter surgical decisions.

**Strengths of the study:** one of the notable strengths of this study is the large sample size collected over 13 years, enhancing the statistical power and relevance of our findings. Additionally, the inclusion of multiple diagnostic variables such as FNA cytology, TIRADS scoring, and tumor size provides a multidimensional perspective reflective of real-world clinical evaluation. The use of standardized ultrasound reporting (TIRADS) and correlation analyses strengthens the reliability of associations presented.

**Limitations:** several limitations should be considered. First, as a retrospective cross-sectional, single-center study, the findings may not be generalizable to broader populations or healthcare systems with different diagnostic protocols. Second, the use of the coding term “LN = 9” to represent extensive nodal disease is institution-specific and may not align with standardized lymph node staging systems. Third, variability in ultrasound interpretation and FNA sampling techniques introduces a risk of observer bias. Finally, because of the cross-sectional design, long-term outcomes such as recurrence rates and disease-specific survival could not be assessed, which limits the prognostic conclusions that can be drawn from this dataset.

**Clinical implications and future directions:** this study reinforces the importance of a risk-adapted, personalized approach to thyroid cancer surgery. The integration of imaging, cytology, and tumor characteristics offers a structured framework to guide decisions on CND, potentially improving oncologic outcomes while minimizing surgical morbidity. Future research should include prospective, multicenter studies with long-term follow-up and consideration of molecular markers such as BRAF or TERT mutations. The incorporation of recurrence and survival outcomes will further refine predictive models and help establish standardized CND criteria applicable across diverse practice settings.

## Conclusion

This study demonstrated that lymph node involvement remains the most decisive factor in guiding central neck dissection (CND) in patients with thyroid cancer. Tumor size and TIRADS classification also showed moderate but clinically meaningful associations with the decision to perform CND, supporting their roles as complementary risk stratification tools. Although age exhibited a weak correlation, neither sex nor body mass index significantly influenced surgical decisions in this cohort. The integration of tumor characteristics, ultrasound-based TIRADS assessment, and cytologic findings provides a more nuanced, evidence-based framework for surgical planning. These findings reinforce the importance of adopting a personalized approach to thyroid cancer surgery one that minimizes overtreatment while ensuring oncologic adequacy. These findings contribute to the growing body of evidence supporting standardized, risk-adapted surgical decision-making in thyroid oncology. Future prospective studies should incorporate long-term outcomes and molecular risk markers to further optimize patient selection and enhance individualized care strategies in thyroid oncology.

### What is known about this topic

- Tumor size and lymph node involvement are important factors in thyroid cancer prognosis;
- Central neck dissection (CND) remains a debated procedure in clinically node-negative (cN0) patients;
- TIRADS is widely used to stratify thyroid nodules but is not routinely linked with CND decisions.

### What this study adds

- This study reveals that lymph node involvement, tumor size, and TIRADS classification are the most significant predictors of central neck dissection in patients with thyroid cancer;
- Age was only weakly associated with central neck dissection, while sex and BMI had no association;
- About half of the study patients were found to have nodal metastasis, and most thyroid nodules were classified as TIRADS 3-5, highlighting the importance of considering radiologic, pathologic, and clinical factors together in treatment and surgery decision-making.
